# Gondwana’s Apparent Polar Wander Path during the Permian-new insights from South America

**DOI:** 10.1038/s41598-018-25873-z

**Published:** 2018-05-31

**Authors:** Renata N. Tomezzoli, Hugo Tickyj, Augusto E. Rapalini, Leandro C. Gallo, Ernesto O. Cristallini, Guadalupe Arzadún, Farid Chemale

**Affiliations:** 1 Laboratorio de Paleomagnetismo “Daniel A. Valencio”. Instituto de Geociencias Básicas, Aplicadas y Ambientales de Buenos Aires (IGEBA). Consejo Nacional de Investigaciones Científicas y Técnicas (CONICET). Departamento de Ciencias Geológicas, Facultad de Ciencias Exactas y Naturales. Pabellón II (1428), Universidad de Buenos Aires, CABA, Argentina; 2 Departamento de Geología. Facultad de Ciencias Exactas y Naturales, Universidad Nacional de La Pampa. Avda, Uruguay 151, L6300CLB Santa Rosa, La Pampa Argentina; 3 Laboratorio de Termocronología (La.Te Andes). Consejo Nacional de Investigaciones Científicas y Técnicas (CONICET), Las Moreras 310, A4401XBA, Vaqueros, Salta, Argentina; 4 Laboratorio de Modelado Geológico. Instituto de Estudios Andinos (IDEAN). Facultad de Ciencias Exactas y Naturales. Universidad de Buenos Aires, CABA, Argentina; 5 Programa de Pos-Graduaçao em Geologia, Universidade do Vale do Rio dos Sinos, CEP 93022-000 Sao Leopoldo, Rio Grande do Sul Brazil

## Abstract

A long-standing debate regarding the configuration of Pangea during the Late Paleozoic has been going on among the paleomagnetic community concerning the validity of one of two significantly different Pangea reconstructions (Pangea A *vs* Pangea B) since the proposal of Pangea B. Although, Pangea B avoids any continental overlap marring classical Pangea A configuration (Wegener’s type), it requires a Carboniferous-Permian megashear of up to 1500 km to achieve the pre-Jurassic configuration. The existence of this megashear is controversial and has led to a wide range of hypotheses, in order to avoid Pangea A continental overlaps and consequently the need for major intra-Pangea movements and to accommodate the paleomagnetic database within a Pangea A reconstruction. We present paleomagnetic results from Permian volcanic rocks of the El Centinela, La Pampa, Argentina. Undeformed volcanic rocks are not affected by any inclination bias and are, therefore, ideal to test different paleogeographic models. The presence of two different paleopole positions, at the base and the top of the same stratigraphic sequence, makes this location optimal to constrain the track of the Gondwana’s path during the Late Paleozoic, which shows the transition from Pangea B during the Carboniferous-Permian, to Pangea A at the Permian – Triassic boundary.

## Introduction

**Figure 1:**
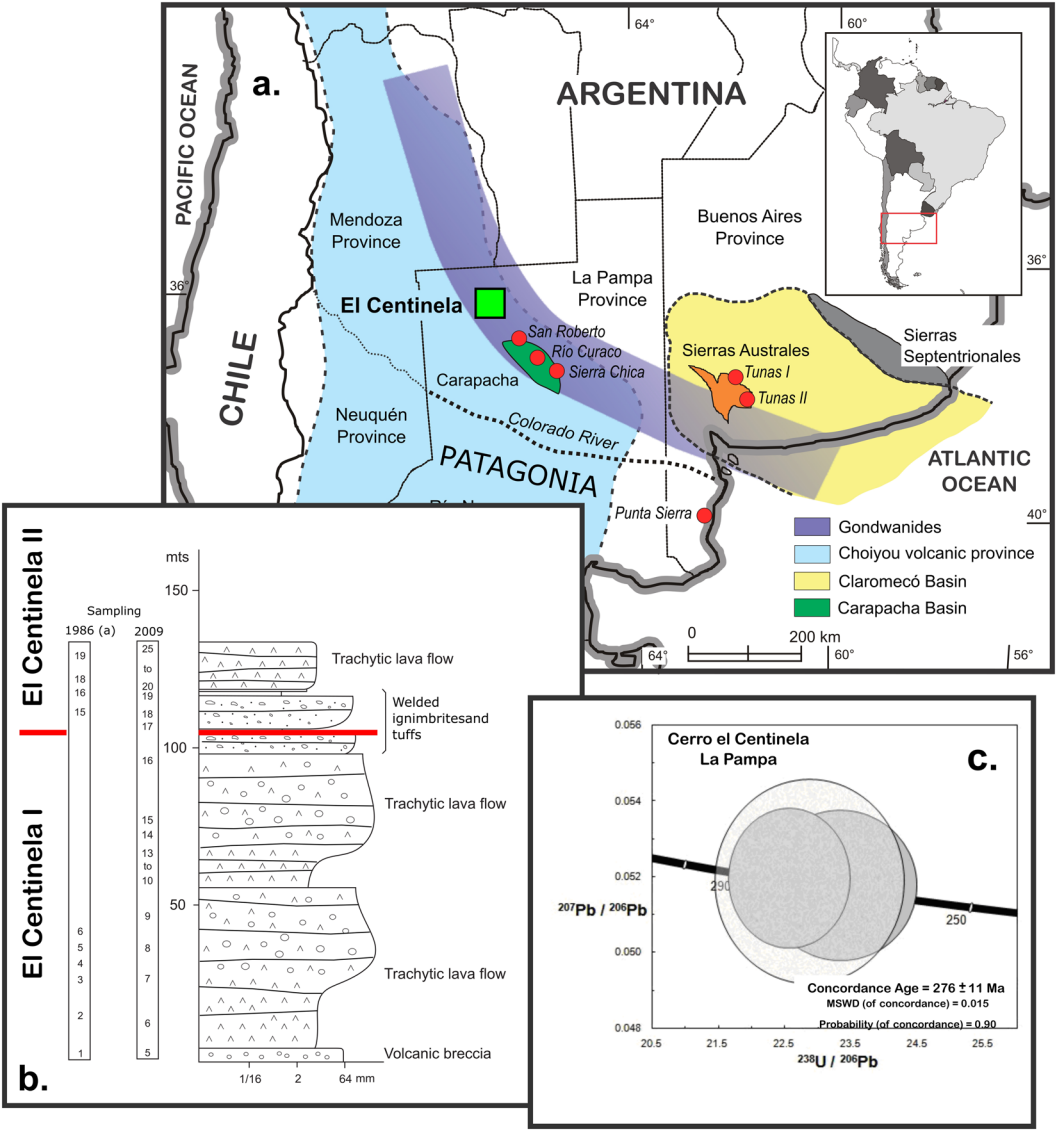
Location and stratigraphic distribution of the paleomagnetic sites and U-Pb age of the top of the Cerro El Centinela. (**a**) The Cerro El Centinela is exposed in the Northwestern of La Pampa province, Argentina, as part of the Gondwanides belt^21^, and other locations with paleomagnetic studies in the surrounding (circles). (**b**) 44 sites in total were sampled from the base to the top of the Cerro El Centinela (see also Table 2) twice: during 1986 (*a* in Table 2) and 2009 respectively. All data were analyzed together. Stratigraphic boundary between both Populations is indicated in the upper part of the sequence (Site CC13a; CC17 respectively). (**c**) U-Pb Tera-Wasserburg plot showing a concordia age of 276 ± 11 Ma (Table 1), which constrains the ending of the magmatic activity to the Kungurian stage in the lower Upper Permian. This figure was digitized from public domain basemaps of South America and Argentina (http://www.ign.gob.ar/AreaServicios/DescargasGratuitas/MapaMudos) and edited with the software Inkscape 0.91 (www.inkscape.org).

The volcanic rocks of the Cerro El Centinela (36°39′S-67°20′W; Fig. 1a) are part of the shoshonitic suite of the Choiyoi Group (Permian-Triassic) in the La Pampa province^1^. They consist of a continuous volcanic sequence of lava flows that degrade to volcanic breccias, interbedded with pyroclastic rocks (Fig. 1b). The set has a homoclinal attitude that changes from Az: 296° to 170°/15°-20° at the base to 175° to 152°/17°-15° to the top of the sequence (bedding plane: strike, 0°-360°, and dip 90° clockwise, from given strike, 0°-90°). The variations in the strike and dip that occur between the different flows must necessarily be of primary origin and are related to the paleotopography of the depositional environment. If these variations were of tectonic origin, the whole volcanic body should have been tilted as a single block and not individually as is observed in the field. Therefore post Permian tectonic deformation can be excluded and the paleomagnetic directions identified considered as being recorded *in situ*.

Despite the fact that zircons are rather rare in ultra potassic volcanic rocks, several attempts have been undertaken in order to collect zircon crystals for radiometric dating. On one of the successful occasions, a sample of 5 kg from the lava flow from the top of the sequence (Fig. 1b,c) was processed and two zircon crystals were separated for isotopic analysis. We obtained an age of 276 ± 11 Ma, which allows the top of the volcanic sequence of the Cerro El Centinela to be placed in the Kungurian stage of the lower Upper Permian (Fig. 1c and Table 1). At the base of the sequence at least five attempts were made to search for zircons that proved unsuccessful. Despite this, we will continue to try.

**Table 1:** Analytical data for zircons of the Cerro El Centinela.

Spot number	**Isotope ratios**							Age (Ma)						% Disc	f206	^232^Th/^238^U
	^207^Pb/^235^U	Error (%)	^206^Pb/^238^U	Error (%)	Rho	^207^Pb/^206^Pb	Error (%)	^206^Pb/^238^U	±	^207^Pb/^235^U	±	^207^Pb/^206^Pb	±			
Zr-119-C-III-01	0.30604	4.12	0.04285	3.25	0.79	0.05179	2.54	71	9	271	11	276	7	2	0.0016	1.22
Zr-119-C-III-02	0.31761	3.55	0.04431	2.67	0.75	0.05198	2.34	280	9	280	10	285	7	2	0.0020	2.91

Sample and standard are corrected after Pb and Hg blanks.

^207^Pb/^206^Pb and ^206^Pb/^238^U are corrected after common Pb presence. Common Pb assuming ^206^Pb/^238^U ^207^Pb/^235^U concordant age.

^235^U = 1/137.88*Utotal.

Standard GJ-1.

All errors in the table are calculated 1 sigma (% for isotope ratios, absolute for ages).

**Figure 2:**
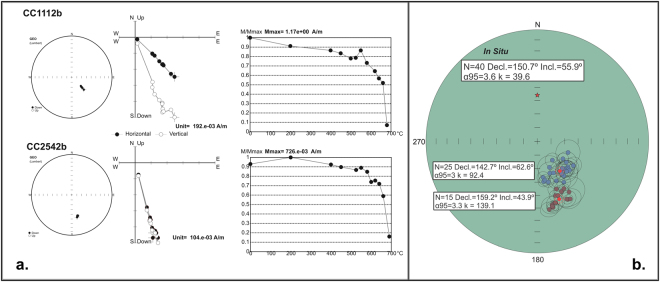
Thermal demagnetization behavior and characteristic remanence directions of the Cerro El Centinela. (**a**) Thermal demagnetization results for selected specimens of the base (CC1112b) and the top (CC2542b) of the sequence from the Cerro El Centinela volcanic rocks, represented as Zijderveld diagrams^2^, cartesian demagnetization curve and equal-area stereographic projection of partial remanence directions (in geographic coordinates). (**b**) Mean site characteristic remanence directions of the Population 1 (base of the sequence, blue circles) and Population 2 (top of the sequence, garnet circles) (in equal area projection) with the respective medias (red circles). See also Table 2. The star indicates the axial dipole field direction. This figure-base was generated with the software Remasoft 3.0. (https://www.agico.com/text/software/remasoft/remasoft.php) and edited with Inkscape 0.91 (www.inkscape.org).

All samples exhibited similar behavior during the progressive thermal demagnetization. They were stable during the early heating steps and started to demagnetize between 600 °C to 680 °C with a gradual quasi-linear or abrupt decay towards the origin^2^ (Fig. 2a). All studied rocks carry a reversed characteristic remanent magnetization (ChRM), with positive (downwards) inclinations (Fig. 2a,b; Table 2) and good within-site directional consistency (α95 < 15° and k > 20), with the exception of sites CC1, CC2, CC4 and CC23 which were not used for further statistical analysis. According to our age determinations, this magnetization was acquired during the Kiaman reverse superchron. The ChRM is carried by hematite product of the oxidation of the magnetite during the cooling of the sequence^3^ suggesting an age of the magnetization coeval with the cooling of the succession. The mean of the ChRM based on 40 accepted sites (Fig. 2b, Table 2) is: Decl. = 150.7°, Incl. = 55.9°, α95 = 3.6° and *k* = 39.6.

**Table 2:** Site mean directions of the characteristic remanent magnetization from the Cerro El Centinela volcanic complex.

*Population I*	N/n	*in situ*		α95°	k
SITE		DEC.º	INCL.º		
CC1 #	6/3	151	61.7	20#	39.2
CC2 #	7/3	126.9	43.6	35#	13
CC3	10/8	131.5	59.3	8	54
CC4 #	7/3	135.4	57.5	27#	89
CC5	5/5	154.4	57.7	6	183
CC6	7/6	158.6	64.9	8	67
CC7	5/5	170.9	63.2	4	318
CC8	8/4	142.4	58	10	82
CC1a	10/10	171	60	4	126
CC2a	9/6	155	69	8.5	63
CC3a	8/8	165	59	4	230
CC4a	8/8	169	61	4	173
CC5a	8/8	153	64	3	275
CC9	7/7	123.9	58	7	99
CC10	6/4	125.9	63.7	8	148
CC11	6/6	133.3	60.7	6	139
CC12	6/6	141.3	62	8	67
CC13	9/8	129.8	60.6	4	176
CC14	7/7	141.3	72.4	5	135
CC15	6/6	133.7	60.9	6.2	118
CC16	8/8	124.3	67.3	4	181
CC6a	8/8	143	60	4	232
CC7a	11/9	147	54	5	105
CC8a	12/12	110	66	9	22
CC9a	10/9	132	58	4	135
CC10a	7/7	136	62	6	114
CC11a	7/7	137	63	4	250
CC12a	5/5	136	59	8	97
***MEAN***	***28***/***25***	***142***.***7***	***62***.***6***	***3***	***92***.***4***
*Population II*		***in situ***			
CC13a	2/2	153	41.5	6.5	—
CC14a	5/4	159	47	3	850
CC17	10/10	161.3	47.5	4	120
CC18	9/8	154.3	48.6	5	120
CC19	6/6	164.5	42.1	2	826
CC15a	9/9	166.5	39	4	160
CC16a	8/8	170	38	6	82
CC20	11/11	152.2	40.9	4	156
CC21	10/8	150.2	40.3	8	54
CC22	8/5	147.8	45.5	11	47
CC23 #	9/6	157.9	60.9	17#	17
CC24	8/6	152.5	49.3	4	313
CC25	9/8	158.1	52.1	6	90
CC17a	8/8	163.5	42	6	91
CC18a	8/8	167	41	7	65
CC19a	8/5	167	40	4	335
***MEAN***	***16***/***15***	***159***.***2***	***43***.***9***	***3***.***3***	***139***.***1***
***in situ***	***44***/***40***	***150***.***7***	***55.9***	***3***.***6***	***39***.***6***

N/n: number of processed specimens/number of specimens used in the calculation of the mean. Dec.: declinations (deg); Inc: inclinations (deg); α95 (deg)=semi-angle of the 95 percent confidence cone; k: Fisher statistical parameters^18^. ^#^Paleomagnetic data from these sites were rejected for the mean; *a*: represents the field sampling carried out in 1986. The sampling sites are ordered stratigraphically from base to the top of the sequence. See also Fig. 1b.

**Figure 3:**
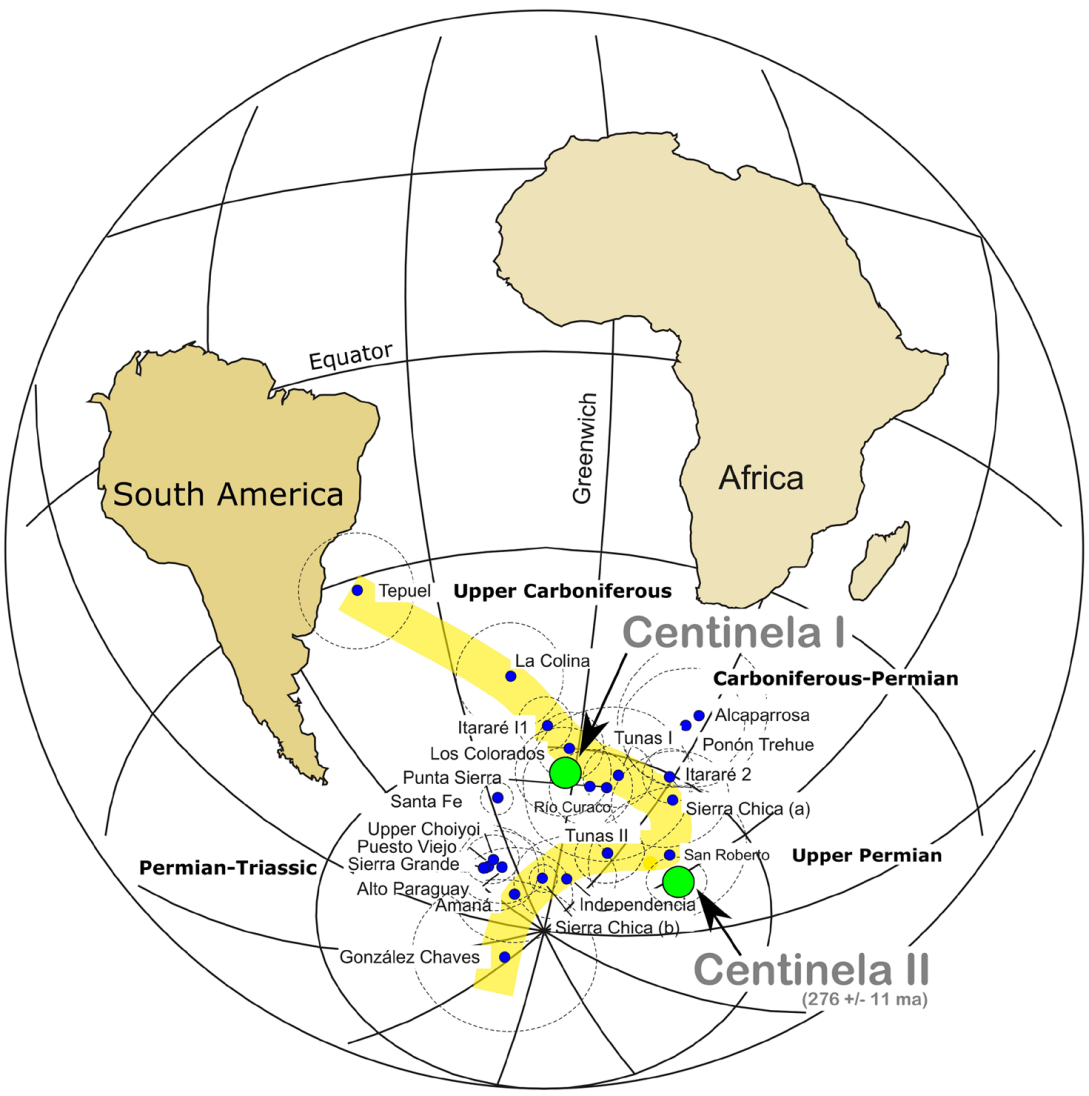
Position of the *El Centinela I* and *El Centinela II* paleomagnetic poles on the Late Paleozoic apparent polar wander path (APWP) for South America^5,6^. This figure-base was generated with the software GMap 2015 (http://www.earthdynamics.org/software/GMAP2015/GMAP.zip) and edited with Inkscape 0.91 (www.inkscape.org).

It is possible to subdivide the ChRM directions into two different populations. The stratigraphic boundary between both populations is located in the upper part of the sequence where the first tuff layer appears at about 100 m above the base (Sites CC13*a*; CC17; Table 2; Figs 1b and 2). The *in situ* mean direction of population 1, is: N = 25, Decl. = 142.7°, Incl. = 62.6°, α95 = 3.0° and *k* = 92.4 (blue circles in Fig. 2b) and for population 2, is: N = 15, Decl. = 159.2°, Incl. = 43.9°, α95 = 3.3° and *k* = 139.1 (garnet circles in Fig. 2b). The great circle distance of 21° of both directions makes them statistically disctinct^4^, indicating that there was enough time between the two populations to average secular variation. Moreover, the internal consistency of each site is very high with alpha 95 lower than 10° (see Table 2) but it is not the same between different sites, demonstrating also that enough time occurred between individual volcanic events. Along the stratigraphic sequence (Fig. 1b), two high quality paleomagnetic poles have been calculated by averaging the virtual geomagnetic poles (VGP) representing each site (Fig. 2b). They are ***El Centinela I*** Paleomagnetic Pole (PP): N = 25, *Lat.: 060.8°S; Long.: 356.6°E*, *A*95 = 4.5*°* and ***El Centinela II*** PP: N = 15, *Lat.: 69.2°S; Long.: 048.2°E*, *A*95 = 3.5° (Fig. 3; Table 2).

Both PPs have good consistency with coeval paleomagnetic poles from others regions of the Southwest Gondwana margin^5,6^ (Figs 1a and 3) with ages bound between the Early Permian (Tunas I PP^7^, with 295.5 ± 8.0 Ma^8^) and the early Late Permian (Tunas II PP^9^, with 280.8 ± 1.9 Ma^10^), Rio Curaco^11^ and San Roberto^11^ PPs, respectively, Sierra Chica (a)^12,13^ PP and Punta Sierra PP^14^. El Centinela I and II PPs have been calculated in volcanic rocks, and furthermore these poles are not the only PPs based on volcanic rocks of South America. The Sierra Chica (a) PP^12^ was also determined in volcanic rocks belonging to the Choiyoi volcanic province^1^, which fully coincides with the age and the position of El Centinela I. Some years later, a different paleomagnetic pole has been published for Sierra Chica (b) PP^15^. Although when it was performed on the same outcrops, the application of an erroneous structural correction and age interpretation of this data^15^ dislocated this PP position^13^.

Each of the El Centinela’s poles represents a significant stratigraphic thickness of more than 50 m (Fig. 1b). Therefore, because of the stratigraphic separation and because of the age difference between El Centinela I (dated from the coeval Tunas I PP)^7,8^ and El Centinela II PPs is about 15 Ma, the declination difference cannot be attributed to secular variation. Instead the difference in the declinations might be attributed to apparent polar wander (Fig. 3).

**Figure 4:**
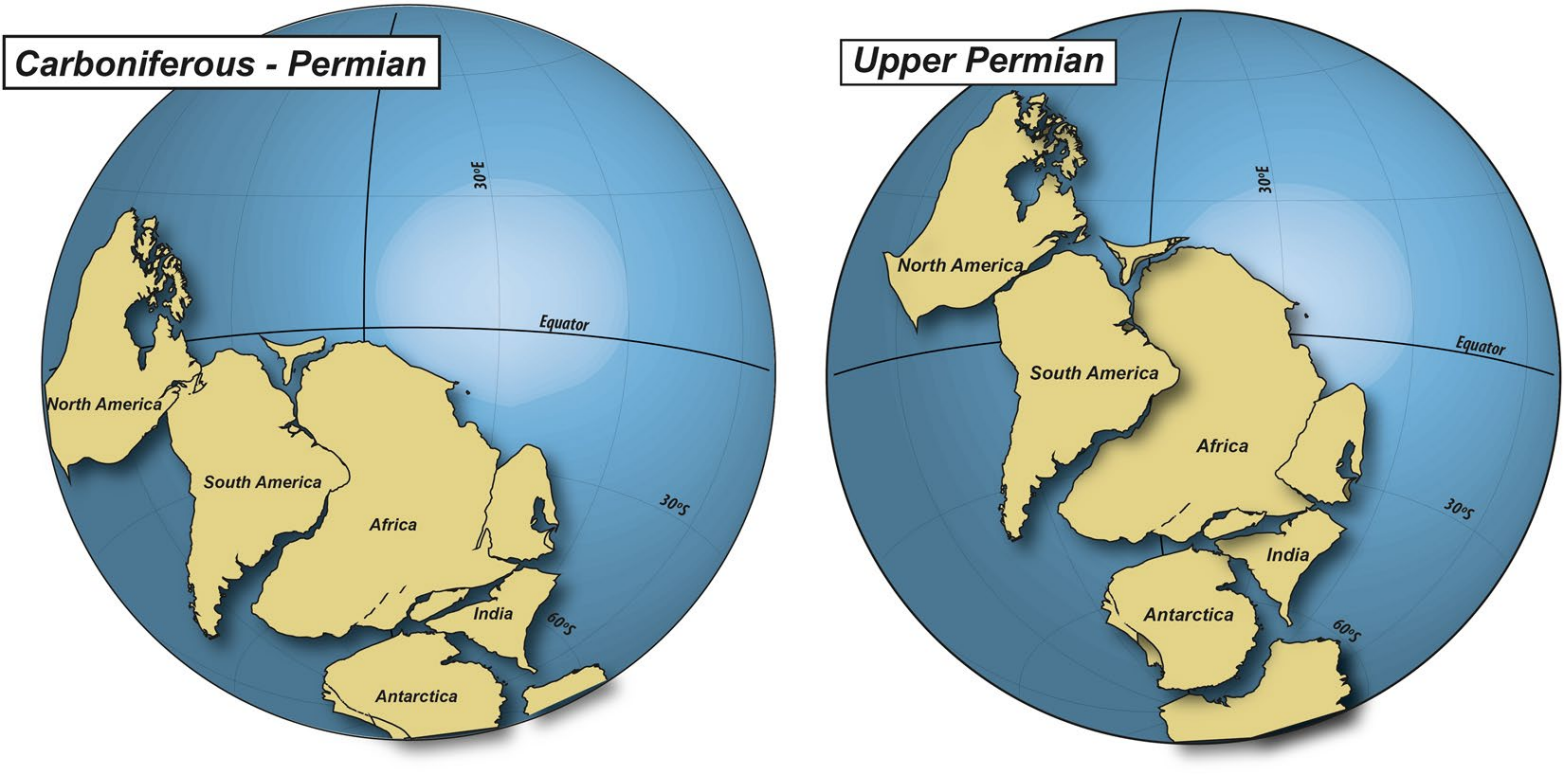
Paleogeographic reconstruction of Gondwana during the Carboniferous-Permian, based on the El Centinela I PP and for the Upper Permian based on the El Centinela II PP. The continents and paleopoles were rotated independently for each time slice^6^. Laurentia reconstruction was performed with poles from igneous and flattening-corrected rocks, selected from the Laurentian database^6^. This figure-base was generated with the software GMap 2015 (http://www.earthdynamics.org/software/GMAP2015/GMAP.zip) and edited with Inkscape 0.91 (www.inkscape.org).

The presence of these two paleopolar positions in the same continuous and undeformed volcanic stratigraphic sequence makes this location perhaps the best example in the world for the study of the paleogeography of Gondwana during the Late Paleozoic. With these poles it is possible to precisely track the APWP for South America during the late Palaeozoic and Triassic and visualize the plate’s movements and the crust related deformation associated with them on the inflections of the APWP^5,6^ (Fig. 3). The continents displacement relative to the geographic South Pole shows the transition from a Pangea B^16^ during the Carboniferous-Permian/Upper Permian (Fig. 4), to a Pangea A in the Permian-Triassic boundary^6^.

## Methods

### Sampling and measurements

A standard paleomagnetic study was carried out from the base to the top of the volcanic succession in two different occasions. The first one was in 1986 where 19 sites were collected with three hand-samples per site. As the magnetic behavior of the samples during demagnetization was very promising (Fig. 2a), a second sampling campaign was carried out in 2009, in which a total of 25 sites (four to six hand samples per site) were collected (Fig. 1b, Table 2).

All samples were oriented in the field using magnetic and sun compasses and an inclinometer; no differences were found between both readings. Measurements of natural remanent magnetization (NRM) were accomplished using a DC squid cryogenic magnetometer (2 G model 750 R) at the “Daniel Valencio” Paleomagnetic laboratory of the Universidad de Buenos Aires IGEBA-CONICET. Thermal demagnetization was the only successful demagnetization method due to the high Curie temperatures of the magnetic carriers, and was applied in at least 15 steps, with maximum temperatures of 680 °C, using ASC ovens with a dual or single chamber. Bulk susceptibility was measured in all specimens after each step to monitor possible chemical changes during heating, with a Bartington MS2 susceptibility meter. Demagnetization results were analyzed using orthogonal vector plots^2^ and stereographic projections (Fig. 2). Paleomagnetic directions were determined using principal component analysis^17^. The final mean directions were computed using Fisher statistics^18^.

The magmatic origin of the zircons was identified through BSE imaging with an electron microscope (JEOL JSM 5800), helped to identify its magmatic origin. Later, they were dated by the U-Pb method using a Laser Ablation Microprobe coupled to a MC-ICP-MS (Neptune), belonging to the Isotope Geology Laboratory of the Federal University of Rio Grande do Sul (Brazil). Isotopic data were acquired using a static mode analysis area of 25 μm in diameter. Calculations were carried out using the Isoplot/Ex 4.10^19^. Instrumental errors^20^ were corrected using the reference zircon GJ-1.
